# Acute abdomen with intraperitoneal free gas: a diagnostic pitfall of pneumatosis cystoides intestinalis—a case report

**DOI:** 10.3389/fmed.2025.1588800

**Published:** 2025-06-30

**Authors:** Hao Wang, Yan-Hui Yang

**Affiliations:** The First Affiliated Hospital, College of Clinical Medicine of Henan University of Science and Technology, Luoyang, China

**Keywords:** pneumatosis cystoides intestinalis, peritonitis, intraperitoneal free air, mixed connective tissue disease, immunosuppression, bacterial translocation

## Abstract

**Background:**

Pneumatosis cystoides intestinalis (PCI) is a rare condition characterized by the presence of multiple gas-filled cysts located beneath the intestinal submucosa or plasma membrane. The pathogenesis of PCI remains incompletely understood, and its clinical presentation lacks specificity.

**Case description:**

A 69-year-old woman was admitted to the hospital with symptoms of abdominal distension, abdominal pain lasting over 2 weeks, and altered consciousness for 3 days. She had a two-year history of mixed connective tissue disease and was undergoing regular hormone therapy. Upon admission, she demonstrated signs of peritoneal irritation, and computed tomography (CT) revealed the presence of multiple free air pockets within the abdominal cavity, as well as “bead-like” translucent areas within the intestinal wall. An emergency abdominal exploration revealed extensive gas accumulation in the wall of the small intestine and multiple gas bubbles in the mesentery. Subsequently, resection and anastomosis of the affected intestinal section were performed. Postoperative pathology identified multiple non-endothelial cystic cavities within the submucosa and muscularis propria of this segment of the small intestine, with *Escherichia coli* and Phocaeicola vulgatus detected in the affected intestinal wall. The patient was further treated in the Intensive Care Unit (ICU) after surgery, but following a brief period of improvement, she developed a lung infection and unfortunately passed away.

**Conclusion:**

This case report demonstrates the diagnostic difficulties in patients with peritonitis with abdominal free gas in intestinal gas cysts, reveals the potential role of abnormal intestinal flora, especially the never reported colonization by Phocaeicola vulgatus, in the development of PCI combined with abdominal free gas.

## Introduction

Pneumatosis cystoides intestinalis (PCI) is a rare condition characterized by the presence of multiple gas-filled cysts within the subplasma or submucosa of the intestine. The etiology and pathogenesis of PCI remain not fully elucidated. Existing literature suggests potential associations with autoimmune diseases (1–3), digestive disorders (4–6), respiratory conditions (7, 8), and various factors, including pharmacological agents (9, 10), hormonal changes, infections (11, 12), and organ transplantation (3, 13). PCI can affect the entire digestive tract, from the esophagus to the rectum, as well as the mesentery and the hepatogastric ligament; however, it predominantly occurs in the small intestine and colon. Clinically, the presentation of PCI lacks specificity, most commonly manifesting as acute abdominal pain. Computed tomography (CT) examinations typically reveal cystic, grapelike, or beaded gas-dense shadows in the affected bowel wall (14–16). Colonoscopy may reveal submucosal white grape-like cysts or red cysts (17–19). When peritonitis is present alongside free gas in the abdominal cavity, differentiating it from gastrointestinal perforation can be challenging (20). Currently, treatment strategies are based on clinical staging, with hyperbaric oxygen therapy (21) and antibiotic intervention utilized for benign forms of PCI, while surgical resection is indicated for severe variants associated with significant complications such as intestinal necrosis, peritonitis, or obstruction (22, 23). This case report details a patient with mixed connective tissue disease who underwent long-term hormone therapy and presented with peritonitis alongside free abdominal gas, initially suspected of gastrointestinal perforation. Intraoperatively, jejunal PCI with characteristic intestinal wall pneumatosis was identified, and pathology confirmed the presence of submucosal multilayered cystic cavities with concurrent colonization by *Escherichia coli* and Phocaeicola vulgatus, leading to death due to a pulmonary infection despite temporary postoperative improvement. This case underscores the diagnostic challenges encountered in patients with peritonitis and free gas within the abdominal cavity, accompanied by intestinal pneumatosis. In clinical practice, the presence of free gas in the abdomen is generally considered a potential indicator of gastrointestinal perforation, often necessitating emergency surgical intervention. Improving clinicians’ understanding of the implications of PCI and free gas in the abdomen can significantly benefit these patients. Furthermore, the bacterial findings in this case, particularly the previously unreported Phocaeicola vulgatus, underscore its potential role in the development of PCI associated with free gas in the abdomen.

## Case report

The patient was a 69-year-old female who presented with abdominal distension and pain persisting for 2 weeks. She experienced abdominal distension, nausea, and vomiting following methylprednisolone treatment for mixed connective tissue disease (MCTD) administered 2 weeks prior, without any medical intervention at that time. Three days later, her symptoms progressed to include loss of appetite and severe vomiting after meals. Gastroscopy conducted at an outside hospital confirmed the diagnosis of fungal esophagitis, with partial symptom relief achieved through mosapride treatment. Subsequently, her abdominal distension worsened, becoming persistent and associated with fatigue, which rendered her bedridden for an extended period. During this time, she experienced a transient fever, peaking at 38.5°C, and her temperature returned to normal following self-medication to reduce the fever. Three days prior to her admission to our hospital, she exhibited blurred consciousness and progressive abdominal distension, and was limited to consuming only small amounts of fluid, prompting her transfer to our facility.

The patient had a history of hypertension, diabetes mellitus, cerebral infarction, and myocardial infarction, and was receiving regular oral antihypertensive and hypoglycemic medications. The patient was diagnosed with mixed connective tissue (MCTD) disease accompanied by a small amount of pericardial effusion 2 years ago and was treated with prednisone acetate. One year ago, the patient experienced an increase in pericardial effusion and subsequently underwent pericardiocentesis, during which the medication regimen was regularly reviewed. Three months ago, the patient underwent pericardiocentesis again, at which point the medication was changed to methylprednisolone, which has been administered since that time.

**Figure 1:**
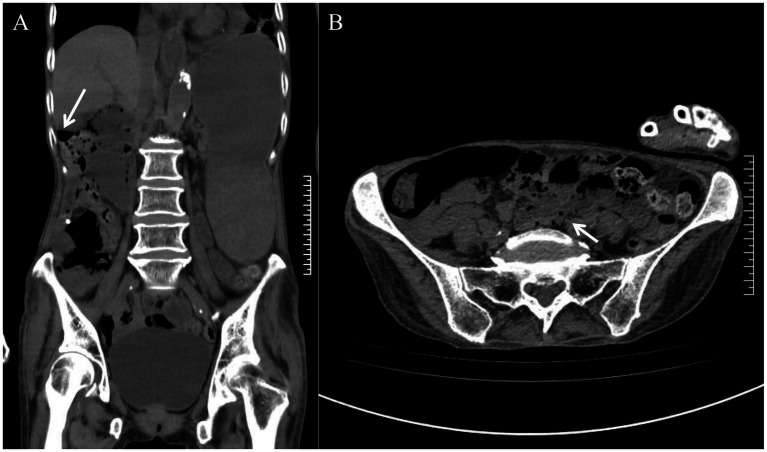
Admission abdominal CT scan. **(A)** An arrow indicates the presence of free gas in the abdominal cavity. **(B)** The arrow indicates multiple translucent gas bubbles within the intestinal wall and mesentery.

The patient was admitted to the hospital exhibiting altered consciousness and apathy. Upon admission, the heart rate was recorded at 121 bpm and the blood pressure was 170/121 mmHg. The examination revealed abdominal distension accompanied by right-sided muscle tension, as well as tenderness with both pressure and rebound pain. Laboratory tests indicated metabolic alkalosis (pH 7.54), mild anemia (erythrocyte count: 3.11 × 10^12^/L; hemoglobin: 103 g/L), and positive occult blood in the gastric fluid, while the leukocyte count remained within normal limits (6.01 × 10^9^/L) and no significant electrolyte abnormalities were observed. Computed tomography (CT) revealed multiple free gas pockets within the abdominal cavity, significant dilation of the gastric cavity, and several translucent gas bubbles in the intestinal wall and mesentery; however, the remaining abdominal parenchyma and retroperitoneum displayed no abnormalities (Figure 1).

**Figure 2:**
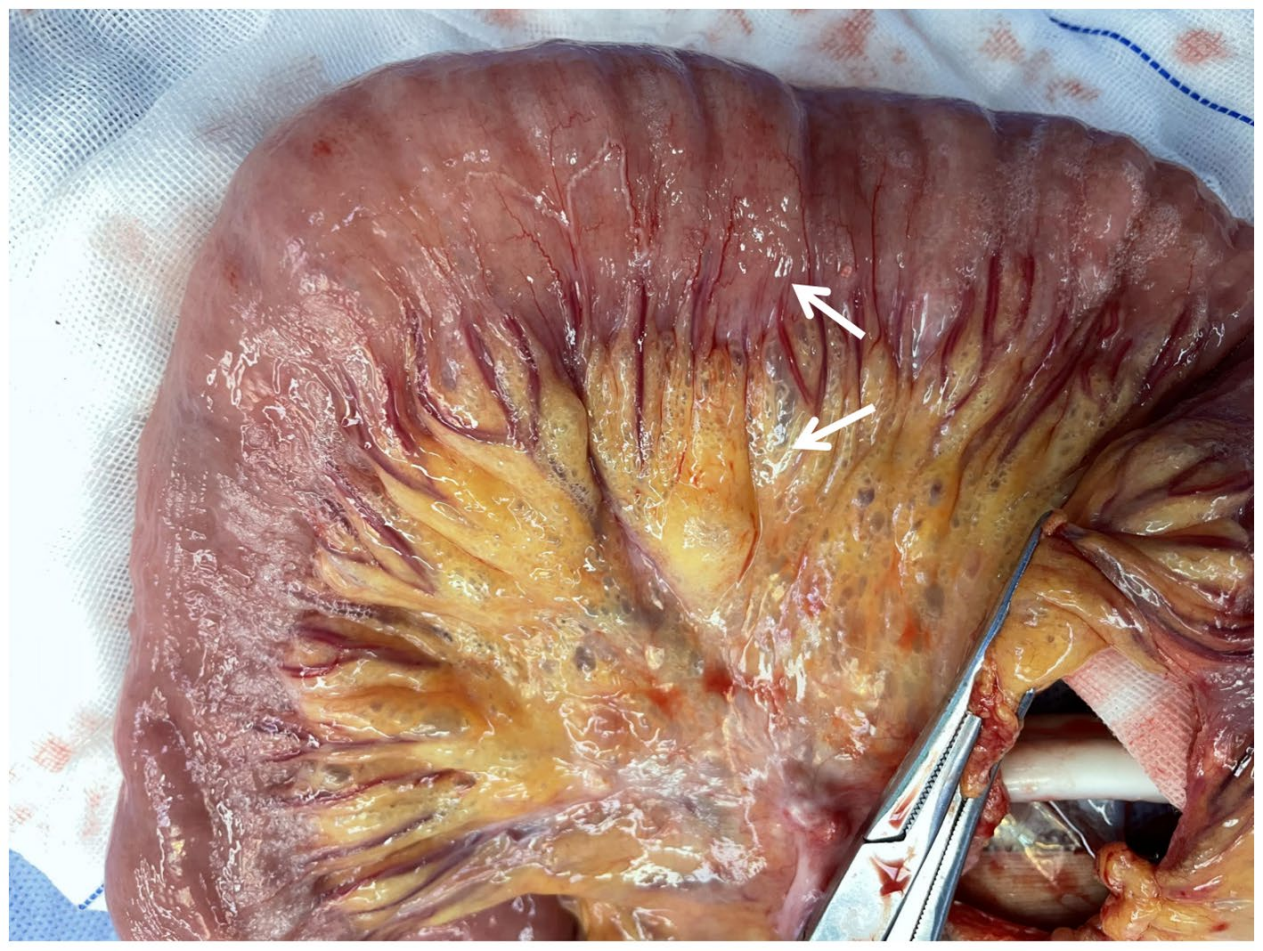
Intraoperative explorations observed. Diseased segment of the small intestine: arrows indicating air bubbles in the intestinal wall and submesenteric region.

**Figure 3:**
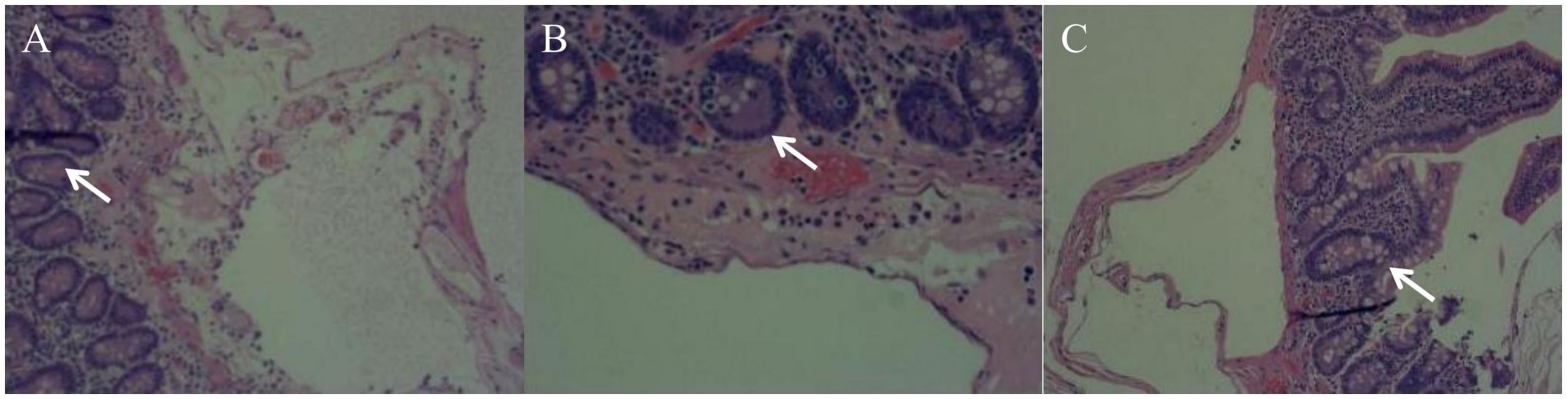
Pathological findings (Multiple pathological sections of the lesion site: **A, B, C**). A segment of the small intestine’s intestinal wall tissue exhibits multiple distended cystic cavities in the submucosal and intermuscular layers; however, no cystic contents are observed.

An emergency laparotomy was performed, revealing significant dilation of the gastric cavity, a soft gastric wall, and no clear evidence of perforation. Approximately 2,000 mL of black-brown gastric contents were evacuated following the combined drainage of a gastric tube. The examination also noted 20 cm of small bowel approximately 110 cm from the flexural ligament, which exhibited redness, swelling, and diffuse pneumatosis, characterized by a “snow-gripping” sensation and firmness with well-defined borders. Multiple scattered translucent air bubbles were observed in the corresponding mesentery, alongside two palpable hard nodules located at the root of the mesentery (Figure 2, 1Supplementary Video 1). A small bowel resection with synchronous *in situ* anastomosis was performed, including resection of the two hard nodules at the mesenteric root. Pathological analysis revealed multiple distended cystic cavities situated between the submucosal layer and the muscularis propria of the small intestine, lacking endothelial or epithelial coverage, and devoid of content; immunohistochemistry ruled out lymphatic vessel or adipose tissue differentiation. The mesenteric and peristomal lymph nodes demonstrated fibrosis with calcification, but neither exhibited tumor components (Figure 3). Microbiological cultures obtained from the diseased intestinal wall indicated abundant colonization by *Escherichia coli* and Phocaeicola vulgatus (Supplementary Video 1).

Following surgery, the patient was transferred to the intensive care unit (ICU) for symptomatic support and treatment with cefoperazone sulbactam sodium as an anti-infective agent. Her conscious status improved, and gastrointestinal symptoms gradually alleviated. On the third day post-surgery, abdominal cultures indicated no infections; however, *Escherichia coli* was detected in the sputum culture. Consequently, the antibiotic was switched to the sensitive agent, imipenem. Gastric fluid tests were negative for occult blood, but the patient developed severe anemia (hemoglobin 62 g/L) along with circulatory disturbances. He showed improvement following several blood transfusions and fluid replacements. A CT scan conducted 6 days after surgery revealed pulmonary exudates, moderate pericardial effusion, and bilateral pleural effusions; intestinal distention was significantly reduced compared to prior evaluations, though some areas of the intestinal wall remained edematous. Polymyxin B nebulized inhalation was initiated, and pericardiocentesis with closed chest drainage was performed, while methylprednisolone was continued to manage the primary disease. On the tenth day post-surgery, lung infection worsened. Blood culture results indicated a human staphylococcal infection, suggesting bacteremia, which was accompanied by a progressive increase in blood lactate levels and multiple organ failure, leading to a rapid deterioration of the patient’s condition and limited responsiveness to symptomatic medication. A day later, despite extensive multidisciplinary resuscitation efforts, the patient succumbed to septicemia induced by severe infections (displacement of lung and intestinal flora), accompanied by infective shock and multiple organ failure. The cause of death was not definitively established due to the family’s refusal of an autopsy.

## Discussion

Pneumatosis cystoides intestinalis (PCI) is a rare condition characterized by the presence of multiple gas-containing cysts located beneath the submucosa or plasma membrane of the gastrointestinal tract, which can involve the entire length of the tract from the esophagus to the rectum, as well as adjacent structures. The clinical presentation of PCI lacks specificity, with symptoms that include abdominal pain, distension, diarrhea, and loss of appetite (22). In severe cases, complications may arise, such as intestinal torsion, obstruction, volvulus, or perforation (23). It is important to note that PCI can be associated with free gas in the abdominal cavity, and its imaging findings may be easily mistaken for gastrointestinal perforation. However, the therapeutic approaches for these two conditions differ significantly; PCI is primarily managed through internal medicine interventions (22, 23), while gastrointestinal perforation necessitates urgent surgical repair or localized intestinal segment resection. In the present case, acute abdomen and free gas in the abdominal cavity were the prominent manifestations, leading to a preoperative consideration of gastrointestinal perforation. Ultimately, PCI was confirmed via intraoperative exploration and pathology. This case underscores the need for heightened awareness of PCI as a potential diagnosis in patients presenting with acute abdomen and free gas, particularly when accompanied by high-risk factors such as immune disorders and prolonged corticosteroid use. Therefore, it is essential to expand the differential diagnosis by integrating CT features (e.g., bead-like translucency in the intestinal wall and mesenteric air bubble shadows) and high-risk factors (such as immune disorders and infections) to facilitate early identification of PCI.

Multiple theories have been proposed regarding the pathogenesis of PCI, including the mechanistic theory, mucosal barrier damage (1–3), bacterial gas production (3, 24), and the theory of pulmonary origin (7, 8). This patient exhibits several key factors contributing to the PCI. We propose that local colonization by *Escherichia coli* and Phocaeicola vulgatus, immune suppression resulting from long-term steroid use that damages the intestinal barrier and mucosa, and intestinal motility disorders associated with MCTD or abdominal infection may significantly influence the progression of PCI in this patient. Furthermore, it is noteworthy that, despite obtaining multiple samples from the affected area for bacterial testing, *Escherichia coli* and Phocaeicola vulgatus are prevalent microorganisms within the intestinal microbiota. The presence of multiple microorganisms raises concerns regarding potential sample contamination. Additional research is warranted to elucidate the impact of *Escherichia coli* and Phocaeicola vulgatus colonization on PCI.

It is important to note that the patient had been diagnosed with fungal esophagitis during a gastroscopy prior to admission. This condition may be associated with his history of MCTD and long-term hormone therapy, potentially contributing to the development of PCI. Early detection and treatment of fungal esophagitis could benefit the patient. However, in this case, the source of the infection was not further investigated or treated. Additional studies are required to confirm these findings.

In this instance, PCI was associated with free gas in the abdominal cavity without gastrointestinal perforation. The production of abdominal gas may be linked to mucosal barrier damage—where immunosuppression increases intestinal mucosal permeability, allowing luminal gas to enter the intestinal wall—and to bacterial gas production, which can occur when bacteria invade the intestinal wall due to disruption or impairment of the mucosal barrier, thereby leading to gas accumulation within the intestinal wall.

The diagnosis of PCI with free gas in the abdominal cavity presents significant challenges. Free gas in the abdominal cavity typically suggests gastrointestinal perforation, which necessitates prompt emergency surgical intervention. Consequently, the diagnostic window available to clinicians is often limited. Furthermore, PCI is a relatively rare condition, and many surgeons may lack familiarity with its specific characteristics, hindering accurate diagnosis. To address these challenges, it is essential to enhance awareness of related surgical conditions. Additionally, the rapid advancement of artificial intelligence (AI) technology may facilitate the diagnosis of such rare diseases. By collecting imaging data pertinent to PCI and developing relevant AI diagnostic models (25, 26), surgeons can improve their ability to diagnose and differentiate these conditions (27). This, in turn, aids physicians in making informed medical decisions and potentially delaying invasive treatments, ultimately benefiting a greater number of patients. Previous studies have explored the use of AI technology to analyze the characteristic imaging features of PCI in CT scans to reduce the risk of misdiagnosis (28). However, current research and data on AI-assisted diagnosis of PCI remain limited, necessitating further studies and data collection to advance this field.

The treatment strategy for PCI is often individualized based on the extent of the lesions, the severity of symptoms, and the risk of complications. Asymptomatic or mildly ill patients typically require no intervention or may benefit from conservative treatments, such as hyperbaric oxygen therapy (21, 22, 29), antibiotics to regulate bacterial flora (11, 12), and nutritional support. In contrast, patients presenting with emergent conditions or complications may require surgical resection of affected bowel segments due to intestinal obstruction, perforation, or hemorrhage (23). In this case, the patient presented to the clinic with an “acute abdomen with free gas in the abdominal cavity,” and the indications for surgery were clear. The resection of the diseased intestinal segment effectively alleviated the gastrointestinal symptoms; however, postoperatively, the patient exhibited intestinal flora shifts and a lung infection due to long-term hormone therapy and immunosuppression. Ultimately, sepsis and multiple organ failure ensued, resulting in a poor prognosis.

PCI in conjunction with peritonitis and the presence of free gas within the abdominal cavity is relatively rare and not widely recognized. This rarity is associated with significant diagnostic challenges due to the nonspecific nature of its symptoms. Furthermore, this study is the first to identify a potential role for localized colonization by *Escherichia coli* and Phocaeicola vulgatus in the development of PCI combined with abdominal free gas. We aim for this case report to enhance the understanding of this condition, provide insights for early diagnosis and pathogenesis, and establish a foundation for standardized diagnosis and treatment, ultimately benefiting more patients presenting with similar conditions.

## Conclusion

This case report highlights the diagnostic dilemma faced by patients presenting with intestinal gas cysts alongside symptoms of peritonitis and abdominal free gas. In clinical diagnosis, the presence of free abdominal gas is frequently regarded as an indicator of potential gastrointestinal perforation, with emergency surgery typically being the primary treatment option. Through the presentation of this case, we aim to enhance clinicians’ understanding of PCI and the implications of abdominal free gas, thereby enabling more patients to benefit from timely interventions.

At the same time, the case report also illustrates the potential role of abnormal intestinal flora, particularly the previously unreported colonization of Phocaeicola vulgatus, in the occurrence of PCI associated with abdominal free gas; the precise association and potential mechanisms of action require further investigation, and this case may offer insights for additional research into the etiology of PCI.

## Data Availability

The original contributions presented in the study are included in the article/Supplementary material, further inquiries can be directed to the corresponding author.
